# When does antimicrobial resistance increase bacterial fitness? Effects of dosing, social interactions, and frequency dependence on the benefits of AmpC *β*-lactamases in broth, biofilms, and a gut infection model

**DOI:** 10.1093/evlett/qrae015

**Published:** 2024-04-20

**Authors:** Elitsa Penkova, Ben Raymond

**Affiliations:** European Centre for Envronment and Human Health, Environment and Sustainability Institute, University of Exeter, Penryn, United Kingdom; Department for Ecology and Conservation, University of Exeter, Penryn, United Kingdom

**Keywords:** antibiotic resistance, beta-lactamase, cooperation, *Enterobacter cloacae*, fitness cost, mutant selection window

## Abstract

One of the longstanding puzzles of antimicrobial resistance is why the frequency of resistance persists at intermediate levels. Theoretical explanations for the lack of fixation of resistance include cryptic costs of resistance or negative frequency-dependence but are seldom explored experimentally. *β*-lactamases, which detoxify penicillin-related antibiotics, have well-characterized frequency-dependent dynamics driven by cheating and cooperation. However, bacterial physiology determines whether *β*-lactamases are cooperative, and we know little about the sociality or fitness of *β*-lactamase producers in infections. Moreover, media-based experiments constrain how we measure fitness and ignore important parameters such as infectivity and transmission among hosts. Here, we investigated the fitness effects of broad-spectrum AmpC *β*-lactamases in *Enterobacter cloacae* in broth, biofilms, and gut infections in a model insect. We quantified frequency- and dose-dependent fitness using cefotaxime, a third-generation cephalosporin. We predicted that infection dynamics would be similar to those observed in biofilms, with social protection extending over a wide dose range. We found evidence for the sociality of *β*-lactamases in all contexts with negative frequency-dependent selection, ensuring the persistence of wild-type bacteria, although cooperation was less prevalent in biofilms, contrary to predictions. While competitive fitness in gut infections and broth had similar dynamics, incorporating infectivity into measurements of fitness in infections significantly affected conclusions. Resistant bacteria had reduced infectivity, which limited the fitness benefits of resistance to infections challenged with low antibiotic doses and low initial frequencies of resistance. The fitness of resistant bacteria in more physiologically tolerant states (in biofilms, in infections) could be constrained by the presence of wild-type bacteria, high antibiotic doses, and limited availability of *β*-lactamases. One conclusion is that increased tolerance of *β*-lactams does not necessarily increase selection pressure for resistance. Overall, both cryptic fitness costs and frequency dependence curtailed the fitness benefits of resistance in this study.

## Introduction

The most fundamental aim of evolutionary biologists in the field of antimicrobial resistance is to be able to understand when the frequency of resistance is going to rise or fall. This is easy to state but challenging to achieve, given the diversity of drugs, resistance mechanisms, and environmental contexts. For example, the evolutionary trajectories of resistance genotypes show variations in the absence of selection — some decline rapidly, while others remain stable or increase in frequency (Enne et al., 2005; Lipsitch, 2001; Whittles et al., 2017). On the other hand, wild-type genotypes may persist even under intense selection for resistance (Colijn et al., 2010). Understanding what drives this variation in the evolutionary responses of bacterial lineages in the presence and absence of antibiotics has important implications for antimicrobial stewardship (Andersson & Hughes, 2011; Raymond, 2019).

Some theoretical models predict that, according to the intensity of selection pressure, competitive exclusion should occur (Colijn et al., 2010), resulting in either resistant or wild-type lineages becoming extinct. Yet, empirical data suggest that the coexistence of both resistant and wild-type genotypes is common (Goossens et al., 2005; Núñez-Núñez et al., 2018; Simonsen, 2018). For example, the prevalence of some resistant pathogens, such as *Staphylococcus aureus*, resistant to penicillin, has been maintained at nearly 100%, while others, such as vancomycin-resistant *Enterococcus* species or *Escherichia coli* resistant to sulphonamides, have remained at intermediate frequencies for long periods (Enne et al., 2001; McDonald, 2006). For other groups, for example, *Streptococcus pneumoniae*, a weak response to selection may have arisen through fundamental trade-offs between resistance and the duration of infection (Lehtinen et al., 2017).

One model proposes that within-host social dynamics shape the evolution of resistance and enable the coexistence of both resistant and wild-type strains of non-obligate pathogens via negative frequency-dependent selection (Davies et al., 2019), a powerful type of balancing selection that maintains genetic diversity in natural population by favoring rare alleles over common ones (Brisson, 2018). Experimentally, it has been repeatedly shown that the evolution of resistance to *β*-lactam antibiotics is influenced by group-beneficial characteristics, driven by frequency-dependent selection (Dugatkin et al., 2005). For example, the secretion of antibiotic-deactivating enzymes, such as *β*-lactamases, reduces the effective drug concentration for the entire community, not solely for conspecifics (Brook, 2009; Klümper et al., 2019; Murray et al., 2018). This group-level protective effect allows wild-type cells to act as “cheats”, leading to fitness that is negatively frequency-dependent (Amanatidou et al., 2019; Dimitriu et al., 2019; Dugatkin et al., 2005; Ellis et al., 2007), ultimately preventing the fixation of resistance through increased competition under high initial antibiotic doses. In contrast to simple competitive exclusion, which might occur when mechanisms of resistance are private, social interactions between microbes can lead to the coexistence of both resistant and wild-type genotypes (Vega & Gore, 2014).

Evidence for sociality has previously been observed in engineered strains of *E. coli* in well-mixed liquid cultures in vitro, where cooperative *β*-lactam detoxification is efficient and allows the survival of wild-type genotypes, albeit over a narrow range of antibiotic doses (Amanatidou et al., 2019; Dugatkin et al., 2005; Perlin et al., 2009). However, in simple structured environments (agar plates), the action of *β*-lactams is rapid and bactericidal, and only a tiny proportion of wild types (the viable persisters) can benefit from detoxification by resistant microbes (Medaney et al., 2015). The bactericidal action of *β*-lactams is dependent on active growth (Tuomanen et al., 1986), and subsequent work showed that social protection of wild types was more prevalent in biofilms than on solid media where a high proportion of cells were not actively growing, even if they were not strictly persisters (Amanatidou et al., 2019). Overall, spatial structure, physiology, and environmental conditions can all affect to what extent social detoxification is a significant process in controlled experiments (Amanatidou et al., 2019; Medaney et al., 2015). This begs the question of whether social detoxification of *β*-lactam antibiotics is important in live infections, see Nakayama et al. (2018) for one example in mice. In addition, if social detoxification is prevalent, can it explain the continued coexistence of resistant and wild-type cells under intense selection pressure from diverse *β*-lactam antibiotics?

Moreover, theoretical analyses rightly consider transmission; between-host fitness components; and infection parameters such as residence time, in models of resistance evolution (Lehtinen et al., 2017; Samore et al., 2005; zur Wiesch et al., 2011). Microbial evolutionary biologists, on the other hand, tend to focus on measurements of fitness in laboratory media, using systems that are well-suited for generating large data sets rapidly. Media-based experiments constrain fitness measurements to either growth rate, productivity, or competitive fitness (the ratio of the Malthusian parameters measured in competition experiments). The relationship between these measures of fitness and transmission is poorly understood. For example, while an influential review suggested that fitness costs of resistance are similar in live infections and in broth (Vogwill & Maclean, 2015), this is not universally accepted (Gagneux et al., 2006; Johnson et al., 2005; Manktelow et al., 2020; Thulin et al., 2015). Work leading up to this study suggests that costs of resistance measured using competitive fitness in broth can be comparable to that in insect infections—but only if infections are established artificially with injections (Penkova, 2023). Therefore, a secondary aim of this study was to explore the impact of resistance genes on fitness measurements that take into account host–bacteria interactions, i.e., the ability of bacteria to establish an infection, and then replicate.

Determining whether bacteria in live infections behave more like those in biofilms in vitro, rather than bacteria rapidly dividing in a nutrient-rich broth, is an important question, yet a difficult one to predict the answer to. In terms of the sociality of *β*-lactamases, one possibility is that infections would behave similarly to biofilms, with social protection extending over a wide range of antibiotic doses. Nevertheless, for social traits, bacterial population size is an important consideration as it determines the total amount of public good and, therefore, the opportunities for cheating (Ross-Gillespie et al., 2009; Zhou et al., 2014). Spatial and social structure or low population sizes in infections might, therefore, limit social exploitation (Zhou et al., 2020; Zhou et al., 2014). We tested these ideas in *Enterobacter cloacae,* an opportunistic pathogen belonging to the ESKAPE group, known to form persistent gut infections in both insects and vertebrates (De Oliveira et al., 2020; Manktelow et al., 2020; Matthews et al., 2019). We measured how frequency, context (broth, biofilms grown on polycarbonate membranes, gut infection), and antibiotic dosage (cefotaxime) would affect the fitness consequences of a broad-spectrum *β*-lactam resistance mechanism and hypothesized that gut infections would behave similarly to biofilms, in terms of increasing antibiotic tolerance and facilitating social interactions. We used a spontaneously resistant strain carrying a mutation consistent with constitutive upregulation of AmpC *β*-lactamase: this mode of resistance is typical of clinical *E. cloacae* and widespread in the *Enterobacteriaceae* (Hilty et al., 2013; Jacoby, 2009; Kaneko et al., 2005; Kuga et al., 2000). AmpC *β*-lactamases along with extended-spectrum *β*-lactamases (ESBLs), are the two main types of resistance mechanisms to cephalosporins, including third-generation cephalosporins (Meini et al., 2018).

## Methods

### Bacterial strains

The wild-type *E. cloacae* ancestor strain was isolated from a colony of diamondback moth at the University of Oxford (Matthews et al., 2019). A spontaneous mutant with resistance to cefotaxime (ctx) was isolated by plating out a dense culture on Lysogeny Broth (LB) agar containing ctx at 4 µg/ml. All subsequent cultures used LB or LB agar at 30 °C. The minimum inhibitory concentrations (MIC) of the ancestor and resistant mutant was 0.25 and 256 µg/ml ctx, respectively, and selective concentrations of 4 µg/ml were used to identify resistant genotypes in competition experiments. The resistant mutant was genetically characterized by partial sequencing of the *ampR* gene, which regulates the expression of AmpC *β*-lactamase. PCR used primers ampR_F74 (5ʹ-TGTGCCTGACAAACGGTTAA-3ʹ) and ampR_R1112 (5ʹ-AGCGGTAAAGGGGTCTTCTA-3ʹ), Qiagen HotStarTaq Plus and the following reaction conditions: 95 °C for 5 min; followed by 35 cycles of 95 °C/30 s, 54 °C/30 s, and 72 °C for 30 s. Sequencing of PCR products was carried out by Eurofins. The mutant strain carried a nonsynonymous mutation at bp410 in *ampR*, which would lead to Asp-Ala substitution. Mutations in *ampR* are consistent with constitutive derepression of *ampC* in *E. cloacae* and have been found in clinical isolates (Kaneko et al., 2005).

### Competition experiments in vitro

Competition in LB was carried out in 1 ml volumes in 24-well plates. Wells were inoculated with 10 µl of inocula, sealed, and incubated for 24 hr with shaking at 180 rpm. We used doses that extended to 8× the MIC of our wild-type *E. cloacae*, and treatments were replicated six times. Biofilms were cultured on polycarbonate filter membranes (Whatman Nucleopore Track-Etch Membranes) and placed on antibiotic-free LB agar, as described previously (Amanatidou et al., 2019). Biofilms were allowed to grow for 24 hr before being transferred to fresh LB plates containing different dosing regimens. Biofilms were transferred daily to fresh medium for three days and then disrupted in 10 ml saline by vortexing. Biofilm treatments were replicated at least six times.

### Insect rearing

We used aseptically reared diamondback moth larvae, *Plutella xylostella,* population VLSS, as our model infection host (Zhou et al., 2018). Aseptic rearing uses an autoclaved diet and is based on previously described methods (Raymond et al., 2009) with the following modifications: eggs were sterilized with 10% Distel (Tristel Solutions, Newmarket, UK) for 1 min followed by two washes of 10 s with sterile water. Larval stock populations were also reared in autoclaved Genesis sterilization containers (V. Mueller brand CD0-3C). The aseptic status of insects was confirmed by culture of uninoculated insect homogenates in each experiment as well as periodic PCR amplification of bacterial 16S rRNA genes, using standard primers (27F, 1492R) (Frank et al., 2008).

### Gut colonization, antibiotic susceptibility, and competition experiments in vivo

We hypothesized that if microbes formed persistent slow-growing colonies in the gut, then wild-type bacteria should exhibit much greater tolerance of *β*-lactam antibiotics than bacteria rapidly dividing in nutrient-rich broth. Assessing the dose–response of wild-type bacteria in gut infections also enabled us to identify realistic dosing regimens that could suppress or clear *E. cloacae*.

Inoculation of diet for insect larvae used previously described methods and used surface-sterilized eggs (Matthews et al., 2019; Somerville et al., 2019). Eggs were allowed to hatch on an inoculated medium for 24 hr only to ensure even-aged cohorts of larvae. After 72 hr from initial inoculation, single larvae were transferred to individual chambers on 128-cell bioassay trays (Frontier Agricultural Sciences Inc.) containing an artificial diet. Diet was supplemented with a range of antibiotic doses: 0, 0.25, 1, 4, 16, 64, 256, or 1,024 µg/ml ctx. Uninfected control larvae were transferred to a diet without antibiotics. Each treatment used 16 replicates in two independent experiments. After 4 days of feeding (now at fourth instar), larvae were surface sterilized in 70% ethanol for 30 s and then homogenized in a bead beater (Qiagen TissueLyser II) in 500 µl saline with a 4 mm steel ball at 30 Hz per second for 4 min. Final cell densities were obtained by dilution plating onto nonselective and selective media to obtain final counts and check for the emergence of spontaneous resistance.

Competition experiments on gut infections used the inoculation methods described in the previous paragraph. Infected larvae were transferred to individual chambers in bioassay trays, where they were fed on an artificial diet containing appropriate ctx doses for 4 days. Gut infection treatments used 48 replicates.

Previous work has shown that *E. cloacae* can form persistent gut infections in insect larvae (Matthews et al., 2019; Somerville et al., 2019), suggesting that these bacteria colonize and attach to the intestinal wall. However, gut infections have not been visualized in this system, and here, we wanted to test whether bacteria were attached to the intestinal wall in biofilm-like growth. To visualize gut infections in *P. xylostella*, fourth-instar larvae, inoculated as above, were dissected by teasing out the intestinal tract using fine entomology pins and fixed in methanol for 10 min. Giemsa’s staining solution (20%) (Fisher Scientific, Loughborough, UK) was applied for 30 min and rinsed in phosphate-buffered saline. Microscopy images were taken using a 60× magnification under bright field illumination with an Olympus BX61. Uninfected larvae were also stained as negative controls.

### Measurements of fitness

In order to test how context, frequency of resistance, and dose affected bacterial fitness, we set up competition experiments in broth, biofilms, and gut infections of our model host. Initial inocula (starting cultures) were prepared by mixing diluted overnight cultures (as above) to give ratios of 1:9, 1:1, and 9:1 of wild-type and resistant bacteria. The wild-type strain was also included as a single-genotype treatment. Initial and final mixtures were plated on both selective and nonselective LB agar and incubated overnight.

**Figure 1.**
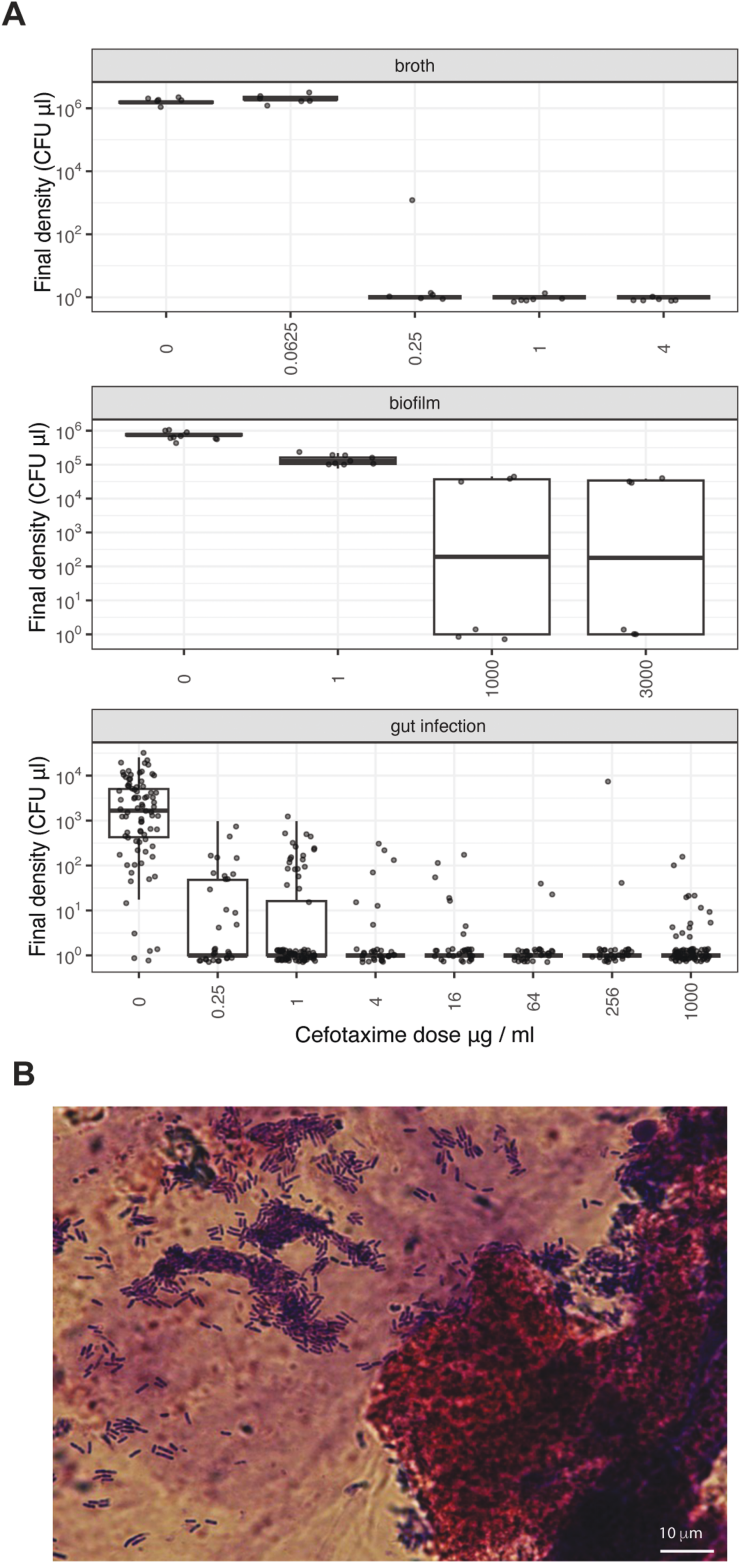
Dose–response of wild-type cefotaxime susceptible *E. cloacae* in three different environmental contexts: nutrient-rich broth (LB), membrane-grown biofilms on LB agar, and gut infections in an insect model. Data are individual replicates with boxplots showing median and interquartile distances (A). The dissected midgut of the fourth instar larva showed blue Giemsa-stained rods associated with *E. cloacae* infection. The pink coloration derives from the stain’s eosin component, which will bind to basic structures. This is consistent with the peritrophic membrane of the insect’s midgut. (B).

Dose–response experiments confirmed that wild-type *E. cloacae* in biofilms and gut infections were much less susceptible to ctx relative to broth cultures (Figure 1). Antibiotic doses were selected to provide a similar range of physiological challenges in different contexts. For all competition experiments, we used an antibiotic-free control, at least one dose that was not fully lethal; one dose (1 µg/ml) was common to all contexts, and in the more antibiotic-tolerant contexts (gut infections and biofilms), we used a common high dose (1,000 µg/ml) that could clear infection or suppress densities by at least two orders of magnitude relative to antibiotic-free controls. Competition experiments in all contexts were run at least twice.

### Statistical analysis

Analyses of bacterial density used log_10_ transformed bacterial densities and doses. In competition experiments, if the estimated density of resistant colonies exceeded the total colony count, wild-type cells were assumed to be extinct. Competitive fitness was calculated as the ratio of the Malthusian parameters (Lenski, 1991; Travisano & Lenski, 1996) and was log-transformed when appropriate to meet assumptions of homoscedasticity. Initial densities for biofilms were calculated from the initial inocula on nonselective agar. In gut infections, the initial population size was assumed to be 50 cells. The small population size of pathogens in the gut is well supported, and calculations of competitive fitness are relatively insensitive to this parameter (van Leeuwen et al., 2015; Zhou et al., 2020). Ctx doses were fitted as covariates in statistical models unless dose responses were clearly nonlinear, in which case dose was fitted as a factor. Initial frequencies of wild-type cells were fitted as covariates.

Relative fitness in competition has long been used as a gold standard for assessing bacterial fitness in evolutionary ecology. Two genotypes are required to be present at the beginning and end of the experiments. In infections, other fitness parameters will impact transmission (infectivity, production of infectious stages). Moreover, even if starting with a mixed inoculum, the stochastic process of colonizing hosts means that many individuals will be colonized with a single genotype. Here, we also used a measure of fitness that incorporated both infectivity (efficiency in establishing an infection) and competition within hosts after infection. To do this, we used the observed proportion of wild-type cells in each larva as a response variable (arc-sine transformed) and examined how antibiotic dose affected fitness after fitting the initial proportion of wild-type cells as a covariate. We also explored the 95% confidence intervals of the proportion of wild-type cells at the end of experiments in relation to the initial proportion of wild-type cells in the inocula. Statistical tests used generalized linear models in R v1.4.1106 (R Core Team, 2021). Sequential removal of nonsignificant terms was used to produce the minimal adequate model, and a graphical analysis of residuals was used to test assumptions of normality and homoscedasticity (Crawley, 2012).

## Results

### Antibiotic tolerance in gut infections is heterogeneous

This experiment aimed to test the hypothesis that antibiotic (ctx) tolerance in gut infections would be more similar to cells grown in biofilms than to cells in broth. In broth experiments, we confirmed our previous MIC measurements, i.e., doses of 0.25 µg/ml were sufficient to prevent the growth of wild-type *E. cloacae* when grown in a monoculture in nearly all replicates, with no growth observed at doses of 1 and 4 µg/ml (Figure 1A). In contrast, much higher doses in biofilms and gut infections were required to suppress bacterial populations. In biofilms, a dose of 1 µg/ml suppressed wild-type growth (reducing densities by roughly an order of magnitude) but was not lethal. Increasing the dose to 1,000 and 3,000 µg/ml was lethal to all cells in half of the replicates and strongly suppressive in other replicates (reductions >2 orders of magnitude) (Figure 1A).

For gut infections, we saw heterogeneous responses to high ctx doses, similar to those observed in biofilms. A dose of 1,000 µg/ml cleared infections to below detection limits for 67 out of 80 replicates and was strongly suppressive in the remainder. The main difference between gut infections and biofilms was that dose responses in vivo were also heterogeneous at lower doses: at 1 µg/ml, 56 out of 80 infections were cleared, while at 0.25 µg/ml ctx only 50% of larvae had infections cleared.

When we imaged infected larvae, we found biofilm-like growth of bacterial rods in large colonies with multiple layers of cells in places (Figure 1B). No other bacterial species were recovered from these aseptically reared insects, which indicates that these microbes are highly likely to be *E. cloacae.*

### Competitive fitness and social detoxification of *β*-lactams

**Figure 2.**
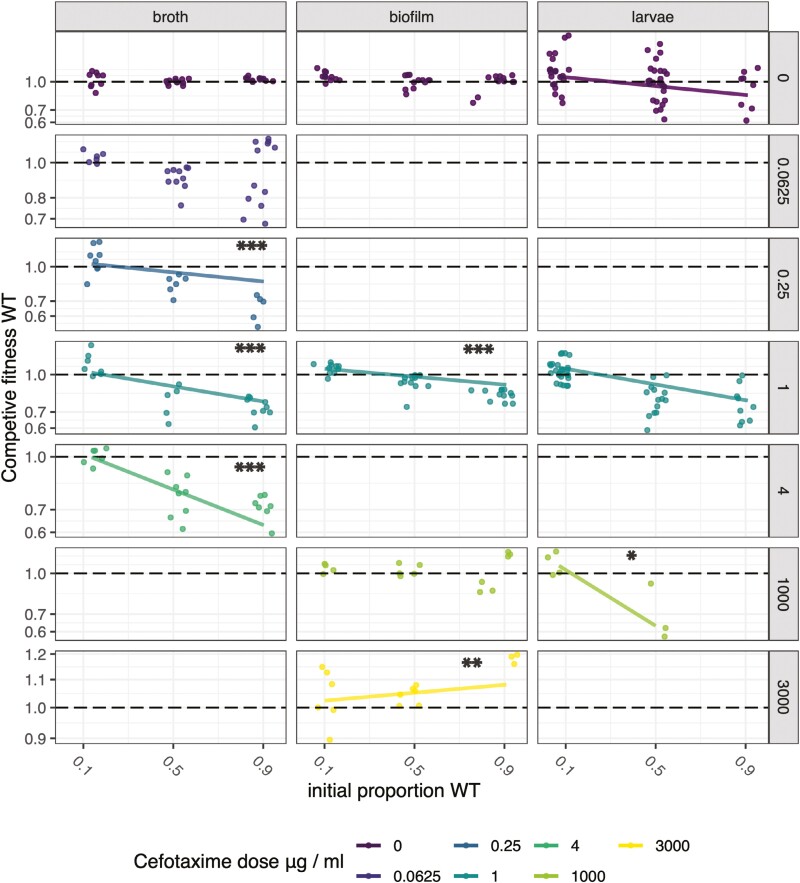
Competitive fitness of wild-type susceptible *E. cloacae* in mixed cultures with resistant genotypes in different experimental contexts. Fitness is calculated as the ratio of the Malthusian parameters. Data points are individual replicates showing frequency-dependent fitness at different doses of antibiotic. Antibiotic doses for each experimental context based on the results presented in Figure 1—we sought to have at least one dose that would be suppressive for susceptible cells and one dose that would be predominantly lethal in each context. Lines are fitted where frequency has a significant effect on fitness in generalized linear models with dose fitted as a factor; asterisks indicate where frequency dependence is significantly different from controls (dose X frequency interactions: *p < 0.05, **p < 0.01, ***p < 0.001).

**Figure 3.**
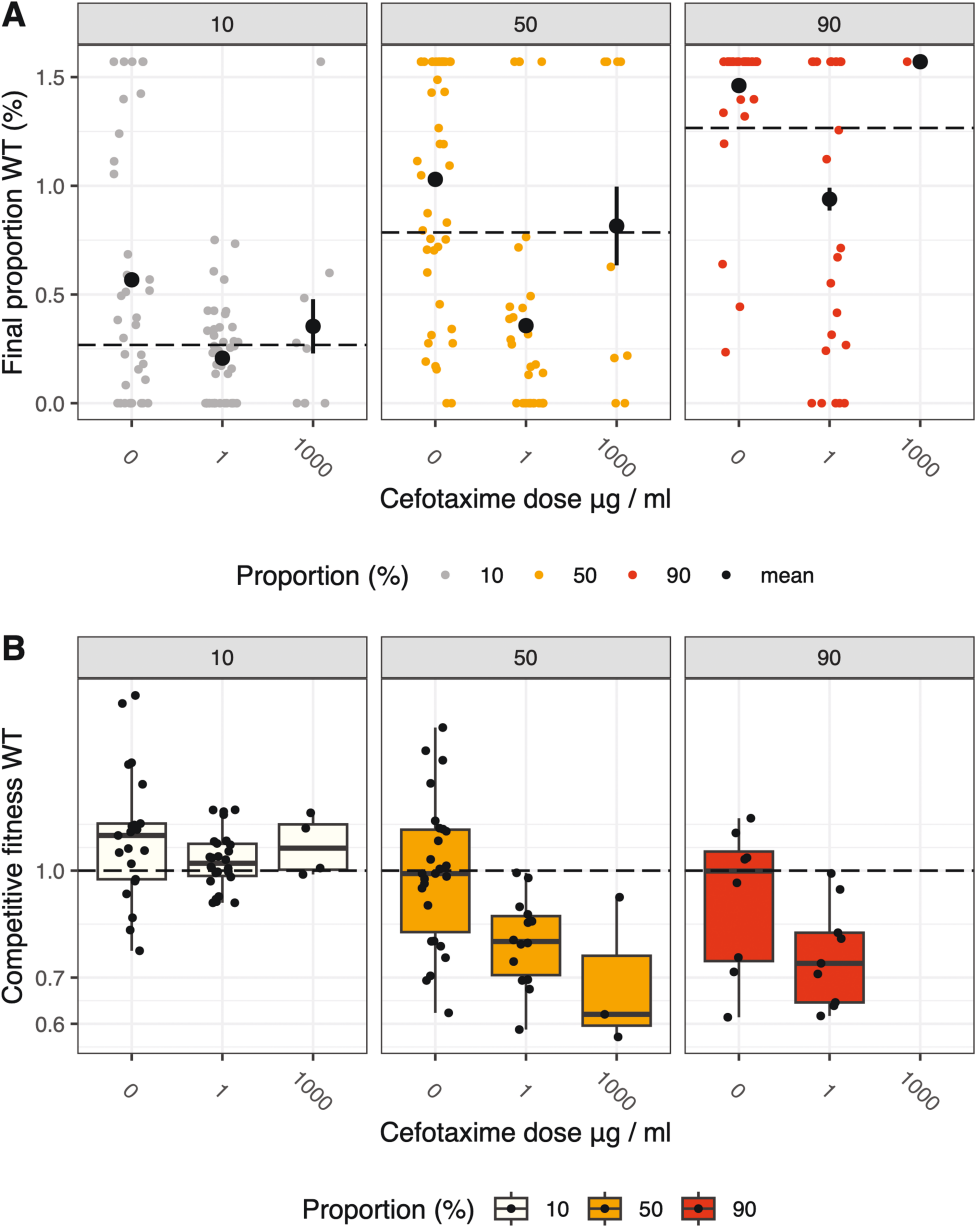
Data showing the overall fitness of wild-type *E. cloacae* (WT) based on the changing patterns in proportion of wild-type bacteria (WT/resistant + WT) in gut infections in an insect model. Dashed lines indicate the initial proportion of wild-type bacteria in the inocula; data points are color-coded by initial proportions in the inocula. Data points are asin-transformed final proportions of wild-type cells after exposure to different doses of antibiotics. Large black circles and error bars show treatment means and 95% confidence intervals. If error bars are not visible, then they are smaller than the data points for the means. If confidence intervals overlap with initial proportions, then wild-type and resistant bacteria have indistinguishable fitness (A). Competitive fitness of wild-type *E. cloacae* in gut infections for comparison with the panel above, with raw data as black circles and boxplots showing medians and interquartile distances (B).

Here, we wanted to test if social protection against cefotaxime shows similar or contrasting patterns in gut infections, biofilms, and broth. We looked for negative frequency-dependent fitness, a pattern consistent with increasing fitness effects due to increased availability of public goods at higher frequencies of resistant bacteria. We also tested for social protection, i.e., whether the presence of resistant cells allows wild-type cells to recover their fitness when exposed to antibiotics. When we analyzed all experimental data together (fitting dose as a covariate), we found a strong three-way interaction between context, antibiotic dose, and wild-type frequency (*F*_2,319_ = 13.5, *p* < 0.0001). There was negative frequency-dependent fitness in all contexts, albeit with a small average effect size (slope −0.078, *SE* = 0.026, *t* = −2.31, *p* = 0.0025) (Figure 2). There was clear social protection of wild-type bacteria in all contexts: antibiotic dose had no effect on fitness in competitions initiated with approximately 10% of wild-type cells (estimate of slope −0.0013, *SE* = 0.0023, *t* = −0.57, *p* = 0.57, Figure 3). There was also no strong evidence of fitness costs associated with resistance across the experiment as a whole (log fitness estimate 0.025, *SE* 0.016, test for difference from zero *t* = 1.59, *p* = 0.11).

In general, at higher frequencies of wild-type cells, cefotaxime could increasingly reduce wild-type fitness (overall dose X frequency interaction estimate −0.11, *SE* = 0.035, *t* = *−*3.20, *p* = 0.0015), although this pattern was not seen in biofilms (dose X frequency interaction estimate = 0.02, test of difference from broth context *t* = 2.37, *p* = 0.019, Figure 2). Competitive fitness showed similar patterns in broth and gut infections: after excluding biofilm data, experimental context had no effect on competitive fitness (*F*_4,225_ = 1.37, *p* = 0.25), both shared the same significant dose X frequency interaction (*F*_1,229_ = 26.0, *p* < 0.0001).

However, there were frequently nonlinear effects of antibiotic dose; therefore, it was sensible to conduct separate analyses for each context, with dose fitted as a factor (Figure 2). In broth, as above, antibiotic dose interacted with wild-type frequency (*F*_4,112_ = 13.9, *p* < 0.0001), and this was driven by significant interactions at the high doses (0.25, 1, and 4 µg/ml, estimates of difference −0.55 *SE* 0.11, −0.47 *SE* 0.11, −0.39 *SE* 0.09, respectively). There was evidence for more effective exploitation of resistant mutants at two intermediate doses (0.25 and 1 µg/ml): intercepts in our statistical model were significantly greater than the antibiotic-free controls, indicating higher overall fitness at these doses (estimates of difference 0.14 *SE* 0.058 and 0.12 *SE* 0.06, respectively). Conversely, WT bacteria could not outcompete resistant mutants at the highest dose of 4 µg/ml.

In biofilms, we also found interactions between antibiotic dose and frequency of wild-type bacteria (*F*_3,90_ = 12.9, *p* < 0.0001). However, strong negative frequency-dependent fitness occurred at the 1 µg/ml dose only (difference estimate −0.22, *SE* = 0.06. *t* = −3.56, *p* = 0.0006). The limited fitness advantages of mutants in biofilms is a striking result here: only at 1 µg/ml did we see the resistant mutant outcompete the wild type; as doses increased, the fitness of WT cells became either similar or greater than that of resistant cells (Figure 2). At the highest dose in biofilms (3,000 µg/ml), there is some evidence of WT fitness increasing with frequency—in contrast to the expectations of negative frequency. However, this positive slope is mostly driven by a few data points at 0.9 WT frequency and the 3,000 µg/ml dose, where low replication resulted from the absence of viable cells in three replicates. The limits on the efficacy of resistance in biofilms exposed to high doses are discussed in more detail below.

Fitness in the gut infection model showed dose-specific results that distinguish it from both biofilm and broth contexts. In gut infections, resistant bacteria could outcompete the wild type at the high dose of 1,000 µg/ml, in contrast to biofilms, but as frequencies declined, wild-type fitness fully recovered, showing efficient social protection at these very high doses, in contrast to broth (Figure 2). To illustrate, competitive fitness at 10% wild-type infections was unaffected by dose (estimate = 0.157, *SE* = 0.11, test of difference from zero *t* = 1.39, *p* = 0.17).

### Infectivity and competitive fitness

A substantial number of gut infections were dominated by single *E. cloacae* genotypes, a pattern consistent with an infection bottleneck. However, our data suggest wild-type bacteria were more prevalent than expected by chance. We assessed how both infectivity and competitive interactions affected the overall fitness of wild-type cells by analyzing the final proportion of the wild type after taking into account the variation in the initial proportions of the inocula. We use the term “overall fitness” to contrast this with competitive fitness.

One key result was that overall fitness did have a simple linear relationship with dose. In gut infections, wild-type bacteria had the lowest fitness at low doses; they were clearly outcompeted by resistant mutants only at 1 µg/ml ctx (dose effect *F*_2,237_ = 29.06 *p* < 0.0001, dose 1 fitness estimate −0.33, *SE* = 0.046; Figure 3A). At the highest doses, in contrast to the analysis of competitive fitness (compare Figure 3A and B), overall fitness was not significantly different from controls (dose 1,000 µg/ml: estimate −0.071, *SE* = 0.086, *p* = 0.43). This result is related to a second key finding: that overall fitness shows much stronger costs associated with resistance in infections. Wild-type cells generally had a greater ability to establish infection than that predicted by a hypothesis of equal fitness, a pattern clearly seen in antibiotic-free controls at all frequencies (Figure 3A).

### Bacterial density and *β*-lactamase limitation

We hypothesized that if bacterial reproduction was limited by the availability of *β*-lactamases, we would see, under antibiotic selection, a declining total bacterial reproduction as the frequency of wild-type cells increased and the availability of *β*-lactamases declined. As our hypothesis relates to the shared benefits of *β*-lactamases, we should see this effect in all bacteria (both resistant and wild-type). There was clear evidence for differences in the extent of *β*-lactamase limitation in the three experimental contexts (broth, biofilms, and insect guts). While it was not possible to analyze all these density data together due to poor adherence to *glm* assumptions, the qualitative differences between the three contexts were strong.

**Figure 4.**
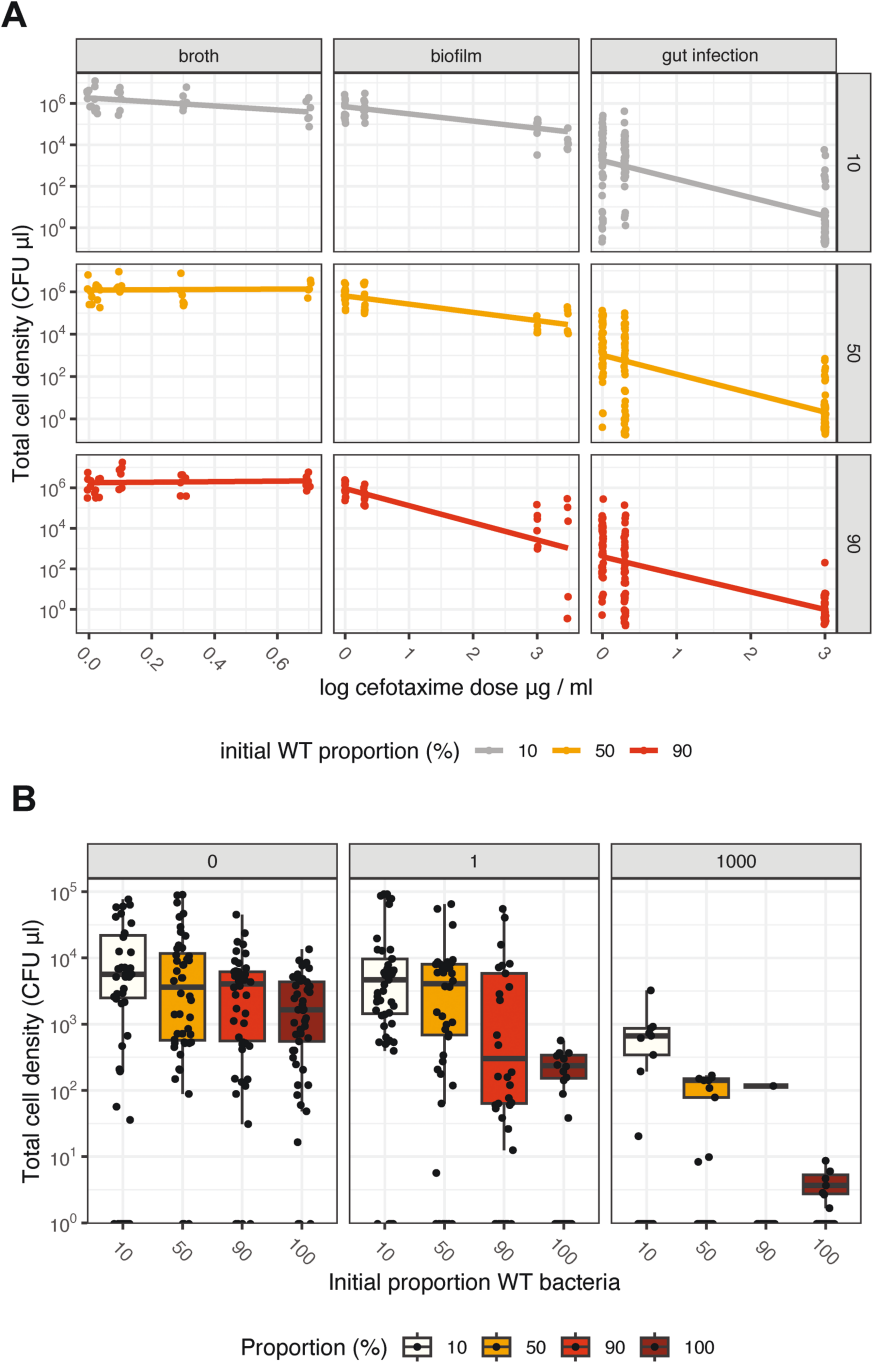
Total bacterial density of *E. cloacae* (WT + resistant bacteria) after exposure to different antibiotic doses in different experimental contexts and at different frequencies of wild-type bacteria. Right-hand panel labels show the initial proportion of wild-type in inocula. Data points are independent repeats, and lines are fitted linear models (A). The total bacterial density in gut infections in insects is the only data from individual animals with box plots showing medians and interquartile distances (B).

In broth experiments, antibiotic dose, and wild-type frequency affected bacterial density (*F*_1,88_ = 9.69, *p* = 0.0025 and *F*_1,87_ = 19.1, *p* < 0.0001, respectively, Figure 4A). However, the effect sizes were very small (antibiotic dose slope −0.252, *SE* = 0.08). Bacterial density also *increased* very slowly with the frequency of wild-type cells (slope 0.0028, *SE* 0.00064), which is not consistent with *β-*lactamase limitation but suggests that growth of resistant cells in broth may have some costs in terms of population size.

In contrast, biofilms showed evidence that *β*-lactamases were limiting at high initial frequencies (90%) of wild-type cells (dose X frequency interaction—*F*_1,104_ = 8.82, *P* = 0.004; Figure 4A). There was also strong evidence for *β*-lactamase limitation in gut infections, as increasing dose affected bacterial population densities at all frequencies of resistant cells (*F*_1,409_ = 360, *p* < 0.0001, Figure 4A). The effects of the dose here were much greater than in broth (slope −0.88, *SE* 0.047, *t* = −18.9, *p* < 0.0001). This is consistent with the overall lower differences in the total density of cells in infections relative to other contexts and the concomitant reduced availability of *β*-lactamases. In gut infections, bacterial growth declined as wild-type frequency increased, a pattern consistent with reduced availability of public goods (*F*_1,408_ = 15.5, *p* < 0.0001).

## Discussion

One of our key aims was to test if social detoxification of a clinically important antibiotic would occur in a live infection model. There was good support for the social protection of wild-type bacteria in gut infections and of negative frequency-dependence generally, consistent with social interactions based on AmpC *β*-lactamases. We are aware of only one other study that has suggested social detoxification occurs in vivo (Nakayama et al., 2018). In addition, most previous studies have focused on less toxic antimicrobials such as ampicillin or carbenicillin (Dugatkin et al., 2005; Frost et al., 2018; Yurtsev et al., 2013, 2016). Previously, we have failed to find evidence for social detoxification of the third-generation cephalosporin cefotaxime by *E. coli* in simple structured environments in vitro (agar plates) at doses just above MIC (Medaney et al., 2015). This is significant, as more rapid killing by more toxic drugs could prevent social protection if local concentrations of antibiotics fluctuate or are not rapidly reduced by the action of *β*-lactamases. There are, however, several differences between these studies that could explain the different results, i.e., the difference in a model organism (*E. coli* vs. *E. cloacae*) and mode of resistance (ESBL vs. derepressed AmpC *β*-lactamase) (Medaney et al., 2015).

We initially predicted that gut infections would be similar to biofilms in terms of increasing tolerance to antibiotics and facilitating social interactions. In several respects, this was true; biofilm-like growth could be identified in the intestine, and similarly high doses (>100 × MIC) were required to effectively suppress growth or clear infection in both biofilms and insects. Here, our microscopy techniques were very straightforward and revealed the presence of bacterial cells in aggregates; further work with confocal microscopy and biofilm component stains is required to confirm the presence of biofilms in vivo. However, there was clearly a proportion of infections that were cleared at much lower MIC equivalent doses, implying a heterogeneous population of bacteria with diverse tolerance to *β*-lactams. As the efficacy of *β*-lactam antibiotics is closely linked to growth rate, these are likely to represent fast- and slow-growing sections of the bacterial population. Heterogeneity in antibiotic tolerance, physiology, and growth rate within human hosts is typical of bacterial pathogens (Kaiser et al., 2014; Mitchison, 1985). However, the competitive fitness of resistant mutants in gut infections resembled broth experiments in some respects, while in other respects (tolerance, overall fitness, and *β*-lactamase limitation), biofilms and gut infections were more similar. One process potentially constraining fitness at high doses was the fact that *β*-lactamases became limiting in biofilms and in gut infections when we used clearly inhibitory doses. With both high doses and *low* frequencies of resistance, there was insufficient production of *β*-lactamases to protect the entire population. Overall survival (for both mutant and wild-type bacteria) was poor, indicating resistance had limited benefits for individuals. In biofilms at high doses, wild-type cells could outcompete resistant mutants, while survival was poor and stochastic at low frequencies of resistance, potentially because other strategies (i.e., tolerance) are now determining survival. In the absence of clear benefits for resistance, the fitness of gut infections at high doses became stochastic even with infections initiated with equal ratios of resistant and wild-type bacteria. In addition, at high doses and *high* frequencies of resistance, population growth was robust, but cheating by the wild type meant that resistant and wild-type bacteria had similar fitness.

A striking result of experiments in biofilms and infections, therefore, was the limited range of doses that provided a competitive advantage to resistant mutants, as we have seen for plasmid-encoded ESBLs in *E. coli* biofilms (Amanatidou et al., 2019). Capturing differences in infectivity for resistant and wild-type bacteria between hosts, rather than just measuring competitive fitness, was important here. The weaker infectivity of mutants curtailed the overall fitness benefits of resistance and contributed to low benefits at all antibiotic doses. It is often assumed that the competitive fitness of bacteria measured in broth translates to fitness impacts in live infections (Vogwill & Maclean, 2015), but this was not the case in this study. The relative growth rate of wild types and resistant mutants was indistinguishable in broth, and so did not predict different fitness in hosts. We note that previous experiments using injections of *Galleria melonella* wax moth larvae, a commonly used insect model, also failed to identify fitness costs and lacked the key step of the natural establishment of infection (Penkova, 2023). Infectivity and the production of infectious material are very important for the dynamics of resistance (Lehtinen et al., 2017; Samore et al., 2005; zur Wiesch et al., 2011), and yet there are few studies quantifying the effects of resistance on transmission even in model hosts (Bogri et al., 2023). Long-term experiments in other animals (cattle) show that a single cephalosporin dose produces only a transient increase in AmpC/ESBL resistance, suggesting fitness costs can return frequencies to low levels (Levent et al., 2022).

The more limited fitness benefits of resistant bacteria in more tolerant contexts contrast with the prevailing view that increased tolerance facilitates the evolution of resistance (Levin-Reisman et al., 2017; Windels et al., 2019a, b). In nutrient-rich media, for example, *E. coli* may evolve greater tolerance prior to acquiring resistance mutations via the regulation of *ampC* (Levin-Reisman et al., 2017). An increased tendency to form highly tolerant persisters has been linked to an increased mutation supply of resistance (Sebastian et al., 2017; Windels et al., 2019a, b). Mutation supply is important for the evolution of resistance in some circumstances, for example, when resistance evolves during infection or when novel modes of resistance first appear (Raymond, 2019; Smith et al., 2002). Nevertheless, mutation supply has limited relevance for resistance to third-generation cephalosporins in commensal bacteria, as these resistance genes are now highly prevalent (Hilty et al., 2013; Kaneko et al., 2005). Selection pressure on resistance, after it has evolved, is a very different process to mutation supply and variation in tolerance captures a large range of physiological states, so it is important not to assume a simple relationship between tolerance and resistance evolution. Early studies showed that periodic exposure of *S. pneumoniae* to high concentrations of penicillin selects for tolerance, while resistant mutants evolve during exposure to low levels of penicillin (Moreillon & Tomasz, 1988), and our results suggest that these strategies may reflect the different relative fitness benefits of resistance at different doses.

The debate over whether high, low, or intermediate doses are best for mitigating the evolution of resistance is still ongoing. It is, therefore, sensible to discuss what a high dose might mean in different contexts. For example, we based experimental doses on measurements of inhibitory action in different contexts, choosing doses that showed effects in broth, biofilms, and insects. However, we can also cross-check our infection doses using rules of thumb used to translate dosing regimens between human and animal models. Typical daily doses of cefotaxime range from 2 to 8 g per day (https://bnf.nice.org.uk/drugs/cefotaxime/) or roughly 0.067 mg kg^−1^ for a 60 kg human. Calculating human equivalent doses uses allometric scaling based on surface area to account for the increasing metabolic rate with smaller size (Nair & Jacob, 2016). Available data on the surface area for small insects, 2–5 mg in our experiments, suggest that doses per unit mass in our system should be around 600 times greater than in humans at 100 µg cefotaxime per insect per day (Kühsel et al., 2017). We estimated daily diet consumption at between 50 and 300 µl per larvae per day, resulting in a cefotaxime dose of between 50 and 300 µg in the high dose treatment of 1,000 µg/ ml diet, which brackets this human equivalent dose. In other words, the “high” dose that was calibrated to be inhibitory in insects matches the human equivalent dose quite well, while the lowest dose, which was only partly inhibitory, was the dose that provided more effective selection for resistance in vivo.

In general, subinhibitory doses can increase mutation supply, but the counterargument against higher doses is that they increase selection pressure (Czuppon et al., 2023; Day & Read, 2016; Kouyos et al., 2014). This study showed the opposite pattern in biofilms and live infections: high doses can actually reduce selection pressure for resistance. This is consistent with more recent improved therapies for *Pseudomonas* infections in cystic fibrosis: delivering high effective doses in the lung through inhaled aerosolized antimicrobials (in contrast to injected antimicrobials) helps mitigate against the evolution of resistance and improves patient outcomes (McCaughey et al., 2013; Moore et al., 2021). In addition, low subinhibitory doses have been shown to effectively select for tetracycline resistance in both laboratory and animal settings (Alexander et al., 2008; Bhattacharya et al., 2017). Effective doses can mitigate against resistance, but on the other hand, overly high doses can increase selection pressure in nontargets (Vasseur et al., 2014). Live infections are complicated and contain bacteria in a range of physiological states (Kaiser et al., 2014; Mitchison, 1985). In addition, a whole range of host metabolites can affect antimicrobial availability or interact with therapies to alter efficacy (Jongmans et al., 2022; Yang et al., 2017). Better data on how dosing and duration of dosing affect the fitness benefits of resistance are needed before generalizations are possible.

## Data Availability

Experimental data and analytical code are available from Zenodo at https://doi.org/10.5281/zenodo.10868386. Mutant and wild-type strains are available upon request from B.R.
